# Predictive value of the admission IBIL/TBIL ratio for poor prognosis after surgical treatment of intracerebral hemorrhage

**DOI:** 10.3389/fneur.2026.1870324

**Published:** 2026-07-28

**Authors:** Song Li, Shirui Wang, Qiang Shao, Wenzheng Li, Fuchao Yu, Xiaowen Zhang, Dianwei Liu, Wei Li

**Affiliations:** 1 Department of Neurovascular Center, Central Hospital Affiliated to Shandong First Medical University and Shandong Academy of Medical Sciences, Jinan, China; 2 Jinan Central Hospital, Shandong University, Jinan, China

**Keywords:** bilirubin ratio, functional outcome, intracerebral hemorrhage, prognosis, restricted cubic spline, surgical evacuation

## Abstract

**Background:**

Serum bilirubin levels are associated with the prognosis of spontaneous intracerebral hemorrhage (sICH). However, the specific predictive value of the indirect-to-total bilirubin (IBIL/TBIL) ratio in patients undergoing surgical hematoma evacuation remains unclear. This study aimed to investigate the association between the admission IBIL/TBIL ratio and 3-month functional outcomes.

**Methods:**

We retrospectively analyzed 168 sICH patients who underwent surgical intervention between January 2024 and December 2025. The primary predictor was the admission IBIL/TBIL ratio. The primary outcome was the 3-month functional outcome, evaluated using the utility-weighted modified Rankin Scale (uw-mRS). Multivariable linear regression and restricted cubic spline (RCS) models were utilized to assess the independent and non-linear associations. Receiver operating characteristic (ROC) curves were generated to compare predictive performances.

**Results:**

Multivariable regression analysis demonstrated that a higher IBIL/TBIL ratio was independently associated with improved functional outcomes [ln(uw-mRS + 1)] after adjusting for age, admission NIHSS score, initial hematoma volume, brain herniation, and secondary intraventricular hemorrhage (overall model *p* < 0.001). The RCS analysis showed an approximately linear relationship between the IBIL/TBIL ratio and functional recovery (*P* non-linearity = 0.568). The IBIL/TBIL ratio showed modest discrimination for 3-month poor functional outcome (mRS 4–6; AUC = 0.655), higher than TBIL alone (AUC = 0.504) but lower than the admission NIHSS score (AUC = 0.784).

**Conclusion:**

The admission IBIL/TBIL ratio is linearly and independently associated with 3-month functional outcome in sICH patients following surgical evacuation. As an inexpensive and routinely available biomarker, it may serve as an adjunctive prognostic indicator, but it should not be interpreted as a standalone replacement for established clinical severity measures.

## 1 Introduction

Spontaneous intracerebral hemorrhage (sICH) remains the most devastating subtype of stroke, accounting for approximately 10 to 15% of all stroke cases worldwide but disproportionately contributing to high rates of mortality and long-term disability (1, 2). Despite advancements in intensive care management, the prognosis for sICH remains poor, primarily due to the primary injury caused by the physical mass effect of the hematoma and the subsequent secondary brain injury (SBI) triggered by heme degradation products (3, 4). Surgical evacuation of the hematoma is a critical intervention aimed at reducing intracranial pressure and mitigating the neurotoxic effects of blood breakdown products (5, 6). However, functional outcomes after surgery remain heterogeneous, necessitating the identification of reliable biomarkers to facilitate early risk stratification and personalized postoperative management.

Following sICH, the lysis of intraparenchymal erythrocytes releases large amounts of hemoglobin, which is catabolized into heme and subsequently degraded by heme oxygenase (HO) into iron, carbon monoxide, and biliverdin. Biliverdin is then rapidly reduced to bilirubin (7, 8). Historically, bilirubin was viewed primarily as a neurotoxic byproduct, particularly at high concentrations where it contributes to cerebral edema and neuronal apoptosis (9). However, emerging evidence has redefined bilirubin as a potent endogenous antioxidant and anti-inflammatory agent (10). At physiological or moderately elevated levels, indirect bilirubin (IBIL) can efficiently scavenge reactive oxygen species (ROS) and inhibit lipid peroxidation, potentially offering neuroprotective effects against the oxidative stress cascade that follows ICH (11, 12).

While many studies have investigated the prognostic value of total bilirubin (TBIL) in stroke, the results have been inconsistent, with some reporting it as a marker of severity and others as a protective factor (13, 14). This discrepancy may stem from the fact that TBIL is a composite of direct and indirect fractions, which have distinct metabolic origins and biological activities (15). IBIL, the primary product of heme catabolism, possesses superior lipophilicity and antioxidant capacity compared to its conjugated counterpart (16). Therefore, the indirect-to-total bilirubin (IBIL/TBIL) ratio may serve as a more specific indicator of the body’s endogenous antioxidant response to the heme load following ICH (17). Focusing on this ratio, rather than total concentration, might bypass the confounding effects of systemic liver function and biliary excretion.

To date, the association between the IBIL/TBIL ratio and functional outcomes in sICH patients undergoing surgical hematoma evacuation has not been thoroughly explored. Furthermore, most prior studies utilized the traditional modified Rankin Scale (mRS), which may lack the sensitivity to detect subtle but clinically meaningful shifts in patient quality of life. In this study, we employed the utility-weighted modified Rankin Scale (uw-mRS) to provide a more nuanced assessment of functional recovery (18, 19, 30, 31). Using restricted cubic spline (RCS) analysis, we aimed to characterize the relationship between the admission IBIL/TBIL ratio and 3-month outcomes across diverse surgical modalities, and to evaluate its predictive performance relative to traditional markers.

## 2 Methods

### 2.1 Study population and design

We conducted a retrospective cohort study of patients with spontaneous intracerebral hemorrhage (sICH) who were admitted to Jinan Central Hospital between January 2024 and December 2025. The inclusion criteria were: (1) diagnosis of sICH confirmed by initial CT scan; (2) age ≥ 18 years; (3) underwent surgical hematoma evacuation. Patients were excluded if they had: (1) secondary ICH (e.g., vascular malformation, trauma, or tumor); (2) pre-existing disability (mRS > 2); (3) severe liver or kidney dysfunction; or (4) incomplete biochemical data or lost to 3-month follow-up. This study was performed in accordance with the Declaration of Helsinki and was approved by the Institutional Review Board and Ethics Committee of the Jinan Central Hospital of Shandong First Medical University (Approval No. 20260127023). Due to the retrospective nature of the study and the use of de-identified data, the requirement for informed consent was waived by the Ethics Committee of the Jinan Central Hospital of Shandong First Medical University.

### 2.2 Surgical procedures

Patients in this cohort received surgical intervention according to hematoma location, hematoma volume, neurological deterioration, mass effect or midline shift on CT, brain herniation or suspected elevated intracranial pressure, intraventricular extension or hydrocephalus when present, general medical condition, and shared decision-making with the patient family or legal representative. Procedures were categorized as minimally invasive surgery (MIS; including robot-assisted or conventional stereotactic puncture, endoscopic evacuation, and ventricular drainage when clinically indicated) or craniotomy for hematoma evacuation. Decompressive craniectomy was recorded separately. For patients undergoing puncture, a drainage catheter was typically left in place for subsequent urokinase irrigation to facilitate residual clot clearance.

### 2.3 Data collection and biomarker measurement

Baseline demographic data (age, sex), clinical severity (admission GCS and NIHSS scores), and imaging characteristics (preoperative hematoma volume, hematoma location, intraventricular hemorrhage, hydrocephalus, brain herniation, and midline shift) were recorded. Surgical modality, decompressive craniectomy, postoperative residual hematoma volume, postoperative rebleeding, hemorrhage progression, 30-day mortality, intracranial infection, pulmonary infection, and deep venous thrombosis were also collected when available. The estimated hematoma clearance rate was calculated as (preoperative hematoma volume - postoperative residual hematoma volume) / preoperative hematoma volume x 100%. Venous blood samples were collected on admission. Serum concentrations of total bilirubin (TBIL) and indirect bilirubin (IBIL) were measured using an automated biochemical analyzer, and the IBIL/TBIL ratio was calculated.

### 2.4 Outcome assessment

The primary outcome was the 3-month functional status, assessed via the utility-weighted modified Rankin Scale (uw-mRS). The uw-mRS assigns weight to each mRS grade based on health-state utilities (e.g., mRS 0 = 1.0, mRS 1 = 0.91, mRS 2 = 0.76, mRS 3 = 0.65, mRS 4 = 0.33, mRS 5 = 0, mRS 6 = 0). The traditional 3-month mRS score was also reported as a clinically familiar reference outcome. For binary analyses, poor prognosis was defined as an mRS score of 4–6.

### 2.5 Statistical analysis

Continuous variables were presented as mean +/− SD or median (IQR). Multivariable linear regression was performed to evaluate the independent association between the IBIL/TBIL ratio and functional outcomes [ln(uw-mRS + 1)], adjusting for age, NIHSS score, hematoma volume, brain herniation, and secondary IVH. Restricted cubic splines (RCS) with four knots were fitted to examine potential non-linear associations, with the *p* value for non-linearity calculated using the Wald test. Receiver operating characteristic (ROC) curves were generated to compare the predictive performance of the IBIL/TBIL ratio, TBIL, and NIHSS score. Postoperative hematoma status, major clinical events, 30-day mortality, and traditional mRS distribution were reported descriptively to improve clinical interpretability. A two-tailed *p* < 0.05 was considered statistically significant.

## 3 Results

### 3.1 Baseline characteristics

A total of 168 patients (mean age 57.9 years; 72.0% male) were included in the final analysis. The median admission NIHSS score was 15, and the median preoperative hematoma volume was 37.0 (19.8–57.2) mL. Regarding surgical interventions, 96 patients (57.1%) underwent MIS and 72 (42.9%) underwent craniotomy. Decompressive craniectomy was performed in 50 patients (29.8%). Postoperative residual hematoma volume was available in 163 patients and was 5.0 (2.0–13.7) mL, with an estimated hematoma clearance rate of 86.3% (31.8–94.8%). Five patients lacked calculable postoperative residual parenchymal hematoma volume. At the 3-month follow-up, the traditional mRS score was 4.0 (3.0–5.0); 47 patients (28.0%) had mRS 0–3 and 121 patients (72.0%) had mRS 4–6.

Postoperative rebleeding was recorded in 18 patients (10.7%). Review of postoperative imaging identified six cases of hemorrhage progression among patients with available documentation. Thirty-day mortality was 10.1% (17/168). Discharge diagnoses indicated intracranial infection in 5 patients (3.0%), pulmonary infection in 73 (43.5%), and deep venous thrombosis in 28 (16.7%). These postoperative events were summarized descriptively because their timing and relationship to functional outcome may differ across patients.

### 3.2 Multivariable analysis of functional outcomes

**Figure 1:**
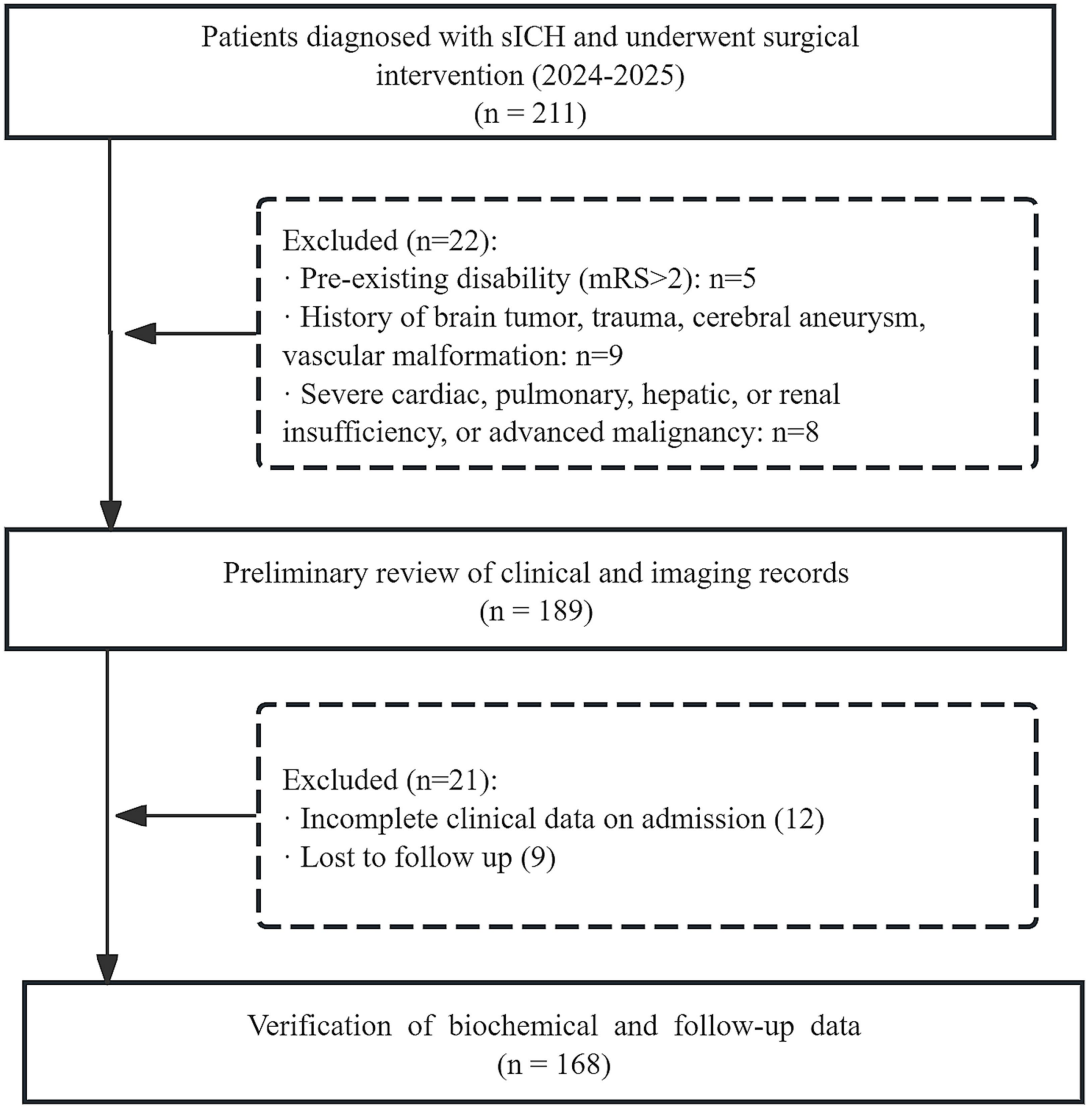
Flowchart of patient selection. A total of 211 patients with spontaneous intracerebral hemorrhage (sICH) received surgical intervention at Jinan Central Hospital between January 2024 and December 2025. After strict screening according to inclusion and exclusion criteria, a final cohort of 168 patients who underwent different surgical procedures was enrolled in this retrospective analysis. The primary outcome was defined as the functional prognosis at the 3-month follow-up. sICH, spontaneous intracerebral hemorrhage; mRS, modified Rankin Scale.

In the multivariable linear regression model, the IBIL/TBIL ratio remained a significant and independent predictor of 3-month uw-mRS scores after adjusting for clinical, imaging, and surgical confounders (Adjusted R^2^ = 0.418, *F* = 15.926, Overall *p* < 0.001). Other significant predictors included the admission NIHSS score (*β* = −0.0173, *p* < 0.001) and age (*β* = −0.0029, *p* = 0.010). The type of surgical modality did not significantly influence the predictive stability of the IBIL/TBIL ratio (Figure 1).

### 3.3 Linear association via restricted cubic splines

**Figure 2:**
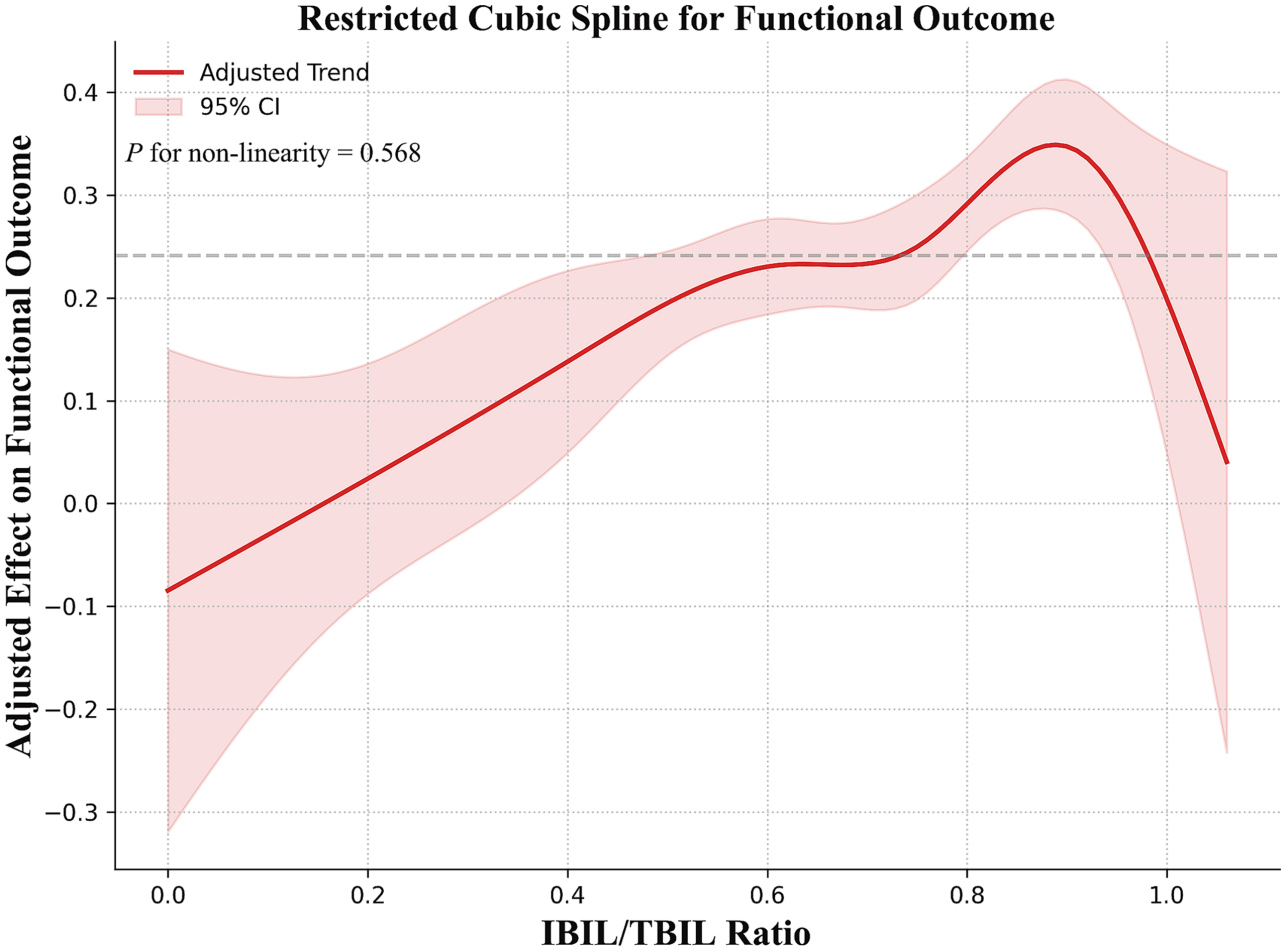
Association between the IBIL/TBIL ratio and functional outcome. The restricted cubic spline (RCS) analysis shows the adjusted relationship between the indirect/total bilirubin (IBIL/TBIL) ratio and the 3-month functional outcome (ln-transformed uw-mRS + 1). The model was adjusted for age, admission NIHSS score, initial hematoma volume, brain herniation, and secondary intraventricular hemorrhage. The solid red line represents the estimated mean effect, and the shaded area indicates the 95% confidence interval. A significant linear association was observed (*p* overall < 0.001, *p* non-linearity = 0.568).

The RCS analysis demonstrated a significant and positive linear relationship between the admission IBIL/TBIL ratio and functional recovery (Table 1). The Wald test confirmed that the association was strictly linear, as no significant non-linear trend was observed (*P* non-linearity = 0.568, Figure 2). As the IBIL/TBIL ratio increased, the adjusted predicted utility weights for the 3-month functional outcome showed a steady improvement (Table 2).

**Table 1:** Baseline characteristics and clinical outcomes of patients stratified by quartiles of the IBIL/TBIL ratio (*N* = 168).

Characteristics	Total	Q 1 (n = 42)	Q 2 (n = 42)	Q 3 (n = 42)	Q 4 (n = 42)	*p* value
IBIL/TBIL range	-	< 0.563	0.563–0.670	0.670–0.795	> 0.795	-
Demographics						
Age (years), mean ± SD	57.9 ± 12.5	56.7 ± 13.9	55.4 ± 10.5	59.1 ± 13.1	60.5 ± 12.1	0.240
Male, n (%)	121 (72.0)	37 (88.1)	30 (71.4)	26 (61.9)	28 (66.7)	**0.044**
Vascular risk factor						
Hypertension, *n* (%)	109 (64.9)	34 (81.0)	23 (54.8)	26 (61.9)	26 (61.9)	0.073
DM, *n* (%)	53 (31.5)	11 (26.2)	12 (28.6)	16 (38.1)	14 (33.3)	0.638
History of stroke, *n* (%)	41 (24.4)	11 (26.2)	9 (21.4)	10 (23.8)	11 (26.2)	0.950
Clinical features						
SBP (mmHg), mean ± SD	157.1 ± 31.8	155.1 ± 31.3	153.2 ± 20.2	159.6 ± 32.1	160.6 ± 37.4	0.650
DBP (mmHg), mean ± SD	90.9 ± 20.2	88.1 ± 17.0	88.6 ± 15.5	92.5 ± 17.7	94.5 ± 28.0	0.397
GCS at admission, median (IQR)	9 (7–11)	9 (7–11)	8 (7–10)	9 (7–12)	9 (8–12)	0.281
NIHSS at admission, median (IQR)	15 (11–19)	15 (11–19)	16 (12–21)	14 (10–18)	14 (10–18)	0.354
Hematoma location 0.344						
- Lobar, *n* (%)	19 (11.3)	7 (16.7)	2 (4.8)	6 (14.3)	4 (9.5)	-
- Deep, *n* (%)	130 (77.4)	29 (69.0)	38 (90.3)	31 (73.8)	32 (76.2)	-
- Infratentorial, *n* (%)	19 (11.3)	6 (14.3)	2 (4.8)	5 (11.9)	6 (14.3)	-
Preoperative hematoma volume (mL), median (IQR)	37.0 (19.8–57.2)	47.0 (18.3–72.0)	36.4 (19.8–52.5)	38.8 (22.8–59.0)	31.0 (19.2–44.5)	0.161
Postoperative residual hematoma volume (mL), median (IQR)	5.0 (2.0–13.7)	9.5 (3.0–18.1)	3.6 (2.7–10.8)	3.2 (2.0–13.2)	3.9 (2.0–15.6)	0.174
Estimated hematoma clearance rate (%), median (IQR)	86.3 (31.8–94.8)	69.8 (1.6–93.4)	87.2 (34.4–94.5)	89.9 (52.3–96.2)	86.5 (29.8–95.5)	0.167
Decompressive craniectomy, n (%)	50 (29.8)	13 (31.0)	9 (21.4)	14 (33.3)	14 (33.3)	0.586
Postoperative rebleeding, n (%)	18 (10.7)	6 (14.3)	2 (4.8)	3 (7.1)	7 (16.7)	0.238
IVH, n (%)	127 (75.6)	31 (73.8)	33 (78.6)	32 (76.2)	31 (73.8)	0.957
Brain Herniation, *n* (%)	21 (12.5)	4 (9.5)	10 (23.8)	2 (4.8)	5 (11.9)	0.056
Surgical method, *n* (%) 0.808						
- MIS, *n* (%)	96 (57.1)	23 (54.8)	26 (61.9)	22 (52.4)	25 (59.5)	-
- Craniotomy, *n* (%)	72 (42.9)	19 (45.2)	16 (38.1)	20 (47.6)	17 (40.5)	-
Total Bilirubin (μmol/L), median (IQR)	17.4 (13.9–21.5)	18.2 (14.5–22.6)	16.5 (13.2–20.4)	17.8 (14.1–21.9)	17.1 (13.8–20.9)	0.512
Indirect Bilirubin (μmol/L), median (IQR)	11.9 (9.2–15.3)	10.4 (8.2–13.5)	11.2 (8.8–14.1)	12.6 (9.5–16.2)	13.5 (10.1–17.4)	**0.008**
IBIL/TBIL ratio, median (IQR)	0.68 (0.59–0.76)	0.54 (0.48–0.58)	0.64 (0.61–0.66)	0.72 (0.69–0.74)	0.81 (0.78–0.85)	**<0.001**
Clinical outcomes						
mRS score, median (IQR)	4 (3–5)	5 (4–6)	4 (3–5)	4 (3–5)	3 (2–4)	**<0.001**
ln (uw-mRS + 1), median (IQR)	0.51 (0.28–0.76)	0.32 (0.00–0.65)	0.50 (0.29–0.78)	0.54 (0.33–0.81)	0.72 (0.51–0.94)	**<0.001**

Data are presented as mean ± SD, median (IQR), or n (%). *p* values were calculated using ANOVA, Kruskal-Wallis H test, or Chi-square test as appropriate. GCS, Glasgow Coma Scale; NIHSS, National Institutes of Health Stroke Scale; IVH, intraventricular hemorrhage; MIS, minimally invasive surgery; IBIL, indirect bilirubin; TBIL, total bilirubin; mRS, modified Rankin Scale; ln (uw-mRS+1), natural logarithm of utility-weighted modified Rankin Scale score plus 1. Bold *p* values indicate statistical significance (*p* < 0.05).

**Table 2:** Univariate and multivariate linear regression analysis of factors associated with 3-month uw-mRS scores (uwmRS was log-transformed as ln(uwmRS + 1) to satisfy normality assumption).

Variables	Univariate *β* (95% CI)	*P* value	Multivariate *β* (95% CI)	*p* value
IBIL/TBIL ratio	0.352 (0.164, 0.541)	**< 0.001**	0.330 (0.174, 0.486)	**< 0.001**
Age (years)	−0.003 (−0.005, 0.000)	0.056	−0.004 (−0.006, −0.001)	**0.002**
Sex (Male)	0.059 (−0.016, 0.135)	0.124	0.040 (−0.022, 0.102)	0.209
Hypertension	−0.042 (−0.116, 0.032)	0.265	−0.054 (−0.112, 0.003)	0.063
Brain herniation	−0.167 (−0.243, −0.091)	**< 0.001**	−0.039 (−0.115, 0.037)	0.314
Admission NIHSS score	−0.019 (−0.024, −0.015)	**< 0.001**	−0.017 (−0.022, −0.012)	**< 0.001**
Initial hematoma volume (mL)	−0.001 (−0.002, 0.000)	**0.007**	−0.000 (−0.001, 0.001)	0.409
Intraventricular hemorrhage	−0.142 (−0.211, −0.074)	**< 0.001**	−0.050 (−0.109, 0.008)	0.092

uw-mRS, utility-weighted modified Rankin Scale; *β*, unstandardized coefficient; CI, confidence interval; IBIL, indirect bilirubin; TBIL, total bilirubin; NIHSS, National Institutes of Health Stroke Scale. The multivariate linear regression model was constructed using the enter method and adjusted for all variables listed in the table. Overall multivariate model: Adjusted R^2^ = 0.418, *F* = 15.926, *p* < 0.001. All variance inflation factors (VIF) < 1.5, indicating no significant multicollinearity. Bold *p* values indicate statistical significance (*p* < 0.05).

### 3.4 Predictive performance and ROC analysis

**Figure 3:**
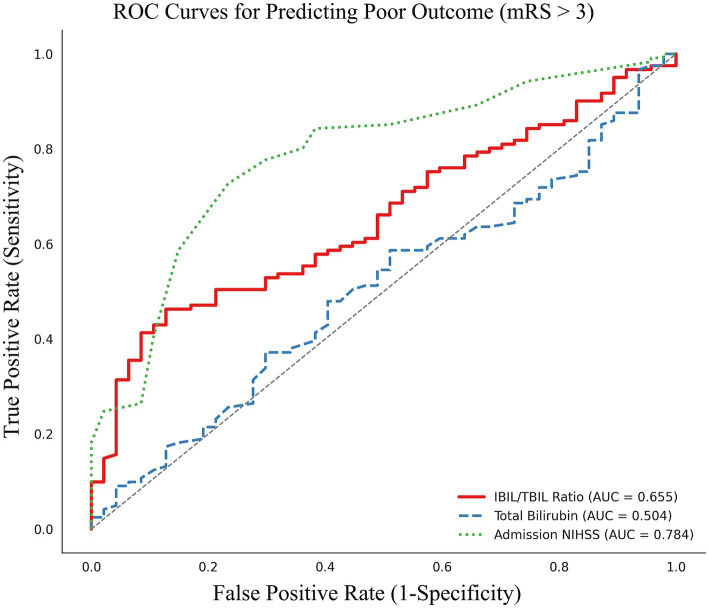
Receiver operating characteristic (ROC) curves for the prediction of 3-month poor functional outcome. The predictive performance of the indirect-to-total bilirubin (IBIL/TBIL) ratio, total bilirubin (TBIL), and admission NIHSS score for predicting poor functional outcome (mRS 4–6) at 3 months was evaluated using ROC curve analysis. The IBIL/TBIL ratio (red solid line) showed modest discrimination (AUC = 0.655), which was higher than TBIL alone (blue dashed line, AUC = 0.504) but lower than the admission NIHSS score (green dotted line, AUC = 0.784). The diagonal grey dashed line represents the line of identity (AUC = 0.500). IBIL, indirect bilirubin; TBIL, total bilirubin; NIHSS, National Institutes of Health Stroke Scale; AUC, area under the curve; mRS, modified Rankin Scale.

ROC curve analysis was performed to evaluate the ability of biomarkers to predict poor functional outcome (mRS 4–6). The IBIL/TBIL ratio exhibited modest predictive capability with an area under the curve (AUC) of 0.655. This performance was higher than that of TBIL alone (AUC = 0.504), which showed little discriminatory value, but lower than that of the admission NIHSS score (AUC = 0.784; Figure 3).

## 4 Discussion

In this retrospective cohort study of 168 patients, we investigated the prognostic significance of the admission indirect-to-total bilirubin (IBIL/TBIL) ratio in patients with spontaneous intracerebral hemorrhage (sICH) who underwent surgical evacuation. A higher IBIL/TBIL ratio was independently associated with improved 3-month functional outcomes, as measured by the utility-weighted modified Rankin Scale (uw-mRS), and the restricted cubic spline (RCS) analysis suggested an approximately linear relationship. However, its discriminatory ability for poor functional outcome was modest and was lower than that of the admission NIHSS score. Therefore, the IBIL/TBIL ratio should be interpreted as a low-cost adjunctive biomarker rather than as a standalone predictor or a replacement for established clinical severity scales.

The role of serum bilirubin in stroke prognosis remains a subject of considerable debate (20). While some studies characterize TBIL as a marker of systemic stress and stroke severity, others highlight its neuroprotective properties (10, 21). This inconsistency likely arises because TBIL comprises both conjugated (direct) and unconjugated (indirect) fractions, each with distinct metabolic pathways and biological activities (9). Direct bilirubin is primarily a marker of hepatobiliary excretion and is influenced by systemic factors, whereas indirect bilirubin (IBIL) is the immediate product of heme degradation (15). By utilizing the IBIL/TBIL ratio, our study minimizes the confounding effects of systemic liver function and isolates the specific metabolic shift of the endogenous antioxidant response (17). This ratio likely represents the proportion of bilirubin that remains actively involved in scavenging reactive oxygen species (ROS) rather than that being cleared by the liver (16).

The association between a higher IBIL/TBIL ratio and favorable outcomes can be explained through the heme oxygenase-1 (HO-1) pathway. Following sICH, the lysis of erythrocytes releases massive amounts of hemoglobin; which is catabolized by HO-1 into biliverdin, iron, and carbon monoxide (7, 16, 22). Biliverdin is subsequently converted into IBIL by biliverdin reductase (23). IBIL is a potent antioxidant that inhibits lipid peroxidation and protects the blood–brain barrier from oxidative stress (24, 25). We hypothesize that a higher admission IBIL/TBIL ratio reflects an efficient and rapid activation of this neuroprotective cascade (12). In patients undergoing surgical evacuation, where the mechanical pressure of the hematoma is relieved, the ability of the brain to neutralize the remaining perihematomal heme-related toxicity becomes a primary determinant of secondary brain injury and functional recovery (26).

Recent clinical trials, such as MISTIE III, have emphasized that the degree of hematoma clearance is linked to functional outcomes (5). In the present cohort, surgical modality and the extent of hematoma removal varied substantially: patients undergoing craniotomy had larger preoperative hematoma volumes and higher estimated clearance rates, whereas MIS was more frequently used for smaller or surgically less accessible hematomas. We therefore recorded surgical category, decompressive craniectomy, postoperative residual hematoma volume, and estimated clearance rate to better characterize this heterogeneity. The prognostic association of the IBIL/TBIL ratio should be considered in this broader perioperative context rather than interpreted independently of surgical decision-making and postoperative course.

Beyond parenchymal hematoma evacuation, intraventricular extension and hydrocephalus introduce additional heterogeneity into surgical management. A recent nomogram identified the modified Graeb score, age, and large infratentorial or thalamic hematomas as predictors of early acute hydrocephalus after intracerebral hemorrhage (27). Evidence does not currently support routine preventive external ventricular drainage for all patients with mild intraventricular hemorrhage (28), underscoring the need to individualize ventricular drainage according to neurological deterioration, hydrocephalus, and ventricular clot burden. Non-vascular structural-related primary intraventricular hemorrhage is also etiologically and prognostically distinct, with admission GCS score, ventricular clot burden, and hydrocephalus associated with outcome (29). These findings support careful characterization of intraventricular hemorrhage and hydrocephalus when interpreting postoperative outcomes in surgically treated intracerebral hemorrhage cohorts.

Several limitations should be considered. First, this was a single-center retrospective study with a relatively small sample size (*n* = 168), which may limit statistical power and generalizability. External validation in larger independent cohorts is required before clinical application can be recommended. Second, we only utilized admission bilirubin levels; postoperative bilirubin was not measured at uniform time points, and dynamic monitoring of the IBIL/TBIL ratio may provide additional prognostic information in future prospective studies. Third, although we added surgical category, decompressive craniectomy, postoperative residual hematoma volume, estimated clearance rate, postoperative rebleeding, major complications, and 30-day mortality, some postoperative variables were retrospectively extracted and their exact timing was not always uniform. Reoperation was not available as a consistently coded variable. Fourth, postoperative clinical events may also lie on the causal pathway between surgery and functional outcome; therefore, they were reported descriptively rather than incorporated into the primary etiologic model. Finally, although secondary intraventricular hemorrhage was included in the multivariable model, ventricular clot burden, acute hydrocephalus, and specific indications for external ventricular drainage were not modeled separately; these features may influence both treatment selection and functional outcome (27–29).

## 5 Conclusion

The admission IBIL/TBIL ratio is linearly and independently associated with 3-month functional recovery in sICH patients undergoing surgical evacuation. Given its modest standalone discrimination and lower AUC than NIHSS, the ratio is best considered a readily available adjunctive biomarker that may complement, rather than replace, established clinical and imaging predictors.

## Data Availability

The original contributions presented in the study are included in the article/supplementary material, further inquiries can be directed to the corresponding author/s.
